# *PKD1*-associated autosomal dominant polycystic kidney disease with glomerular cysts presenting with nephrotic syndrome caused by focal segmental glomerulosclerosis

**DOI:** 10.1186/s12882-019-1524-6

**Published:** 2019-08-28

**Authors:** Yasuhiro Oda, Naoki Sawa, Eiko Hasegawa, Hiroki Mizuno, Masahiro Kawada, Akinari Sekine, Rikako Hiramatsu, Masayuki Yamanouchi, Noriko Hayami, Tatsuya Suwabe, Junichi Hoshino, Kenmei Takaichi, Keiichi Kinowaki, Kenichi Ohashi, Takeshi Fujii, Yoshifumi Ubara

**Affiliations:** 1 Nephrology Center, Toranomon Hospital, 2-2-2 Toranomon, Minato-ku, Tokyo, 105-8470 Japan; 2 Okinaka Memorial Institute for Medical Research, Toranomon Hospital, 2-2-2 Toranomon, Minato-ku, Tokyo, 105-8470 Japan; 3 Department of Pathology, Toranomon Hospital, 2-2-2 Toranomon, Minato-ku, Tokyo, 105-8470 Japan; 4 Department of Pathology, Graduate School of Medicine, Yokohama City University, 3-9 Fukuura, Kanazawa-ku, Yokohama, Kanagawa 236-0004 Japan

**Keywords:** Autosomal dominant polycystic kidney disease, *PKD1*, Glomerular cyst, Focal segmental glomerulosclerosis

## Abstract

**Background:**

Autosomal dominant polycystic kidney disease (ADPKD) may manifest non-nephrotic range proteinuria, but is rarely complicated with nephrotic syndrome. Limited number of reports describe the histology of ADPKD with nephrotic syndrome in detail.

**Case presentation:**

We encountered a 23-year-old man with polycystic kidney disease (PKD) with small kidney volume and nephrotic syndrome, which eventually progressed to end-stage renal disease. Renal histology showed typical focal segmental glomerulosclerosis and remarkable glomerular cyst formation, but did not reveal tubular cysts. *PKD1* mutation was detected in him and his father, who also had PKD with small kidney volume.

**Conclusions:**

In contrast to tubular cysts which develop along ADPKD progression, glomerular cysts may likely be associated with ADPKD with slower volume progression manifesting small kidney volume. Although previous investigations report that ADPKD with smaller kidney volume is attributed to slower decline in renal function, coexistence of nephrotic-range proteinuria implies complication of other glomerular diseases and needs histological evaluation since it may lead to poor renal outcome.

## Background

Autosomal dominant polycystic kidney disease (ADPKD) may present non-nephrotic range proteinuria, generally less than 1 g per day [1], and it is rarely complicated with nephrotic syndrome. Clinical cases of ADPKD with nephrotic syndrome have been reported previously, but their renal pathology is often not described in detail and hence not very well known. Herein, we report a case of a 23-year-old man with *PKD1*-associated ADPKD and nephrotic syndrome, whose renal histology showed typical focal segmental glomerulosclerosis (FSGS) and remarkable glomerular cyst formation. To the best of our knowledge, this is the first reported case of ADPKD with glomerular cyst formation and proven *PKD1* mutation complicated with nephrotic syndrome caused by FSGS.

## Case presentation

A 23-year-old Japanese man was referred to our institution for evaluation of overt proteinuria. Proteinuria was detected through an annual health checkup when he was 13 years old. First renal biopsy was performed at a nearby hospital at the age of 19. Urinary protein excretion was 7.0 g/day; hematuria, negative; serum albumin level, 1.9 g/dL; total cholesterol level, 437 mg/dL; and serum creatinine level, 0.6 mg/dL.

### First renal biopsy

**Fig. 1:**
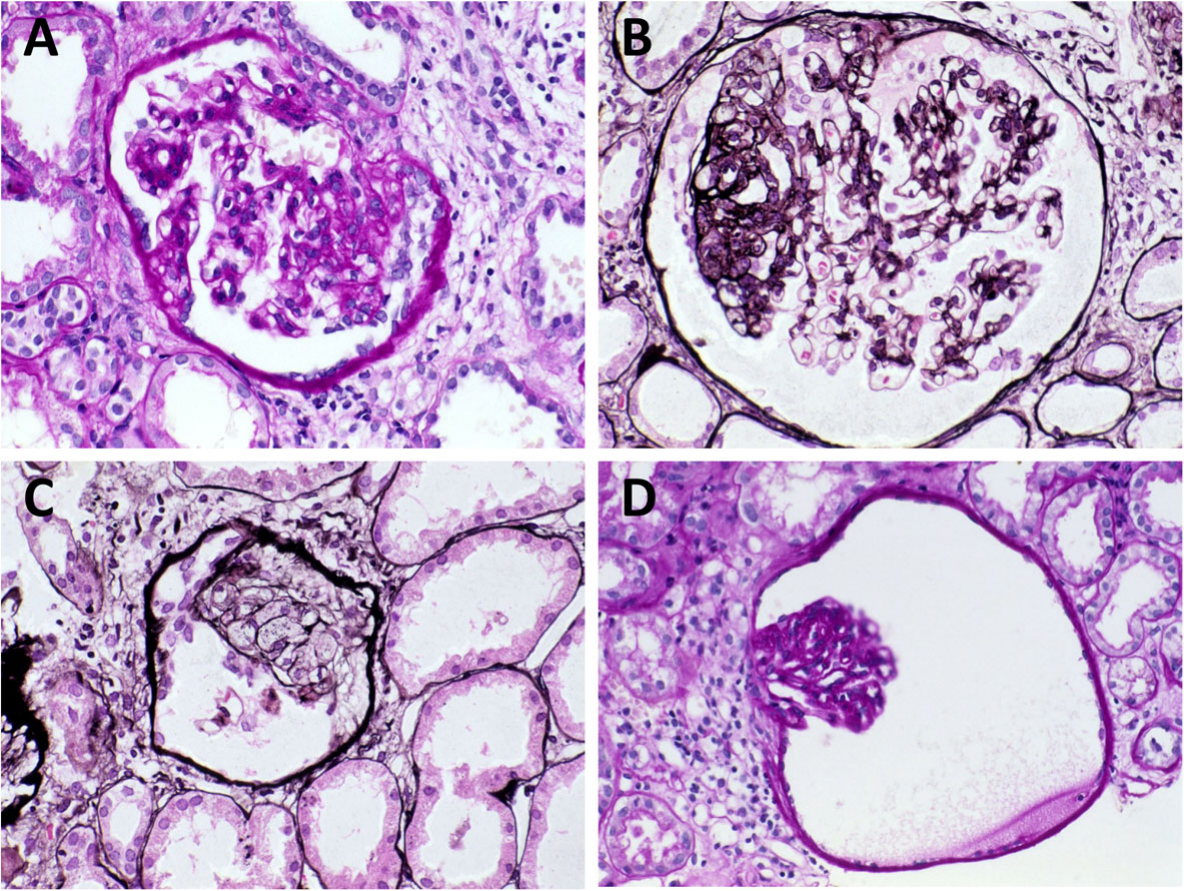
Microscopic findings of the first renal biopsy at the age of 19. Glomeruli have multiple segmental sclerotic lesions (panels **a**, **b**, and **c**: periodic acid-Schiff stain; panel **d**: periodic acid-methenamine-silver stain). Some sclerotic lesions are adherent to the Bowman’s capsule (panels **a**, **b**, and **c**). Collapsed tufts are seen inside widened Bowman’s capsules forming glomerular cysts (panels **c** and **d**)

Renal biopsy revealed segmental sclerosis with foamy change in four out of 13 glomeruli, some of which were adherent to Bowman’s capsules (Fig. 1a, b, and c). No glomerulus had global sclerosis. Several glomeruli had collapsed tufts in widened Bowman’s capsules forming glomerular cysts (Fig. 1c and d). Tubular cysts were not found. Since the histology was compatible with FSGS, prednisolone was started at 40 mg per day. However, urinary protein excretion remained high. Prednisolone was tapered to 5 mg per day. Lower leg edema developed and renal function worsened, and he was referred to our hospital at the age of 23.

### Physical and laboratory examinations

**Fig. 2:**
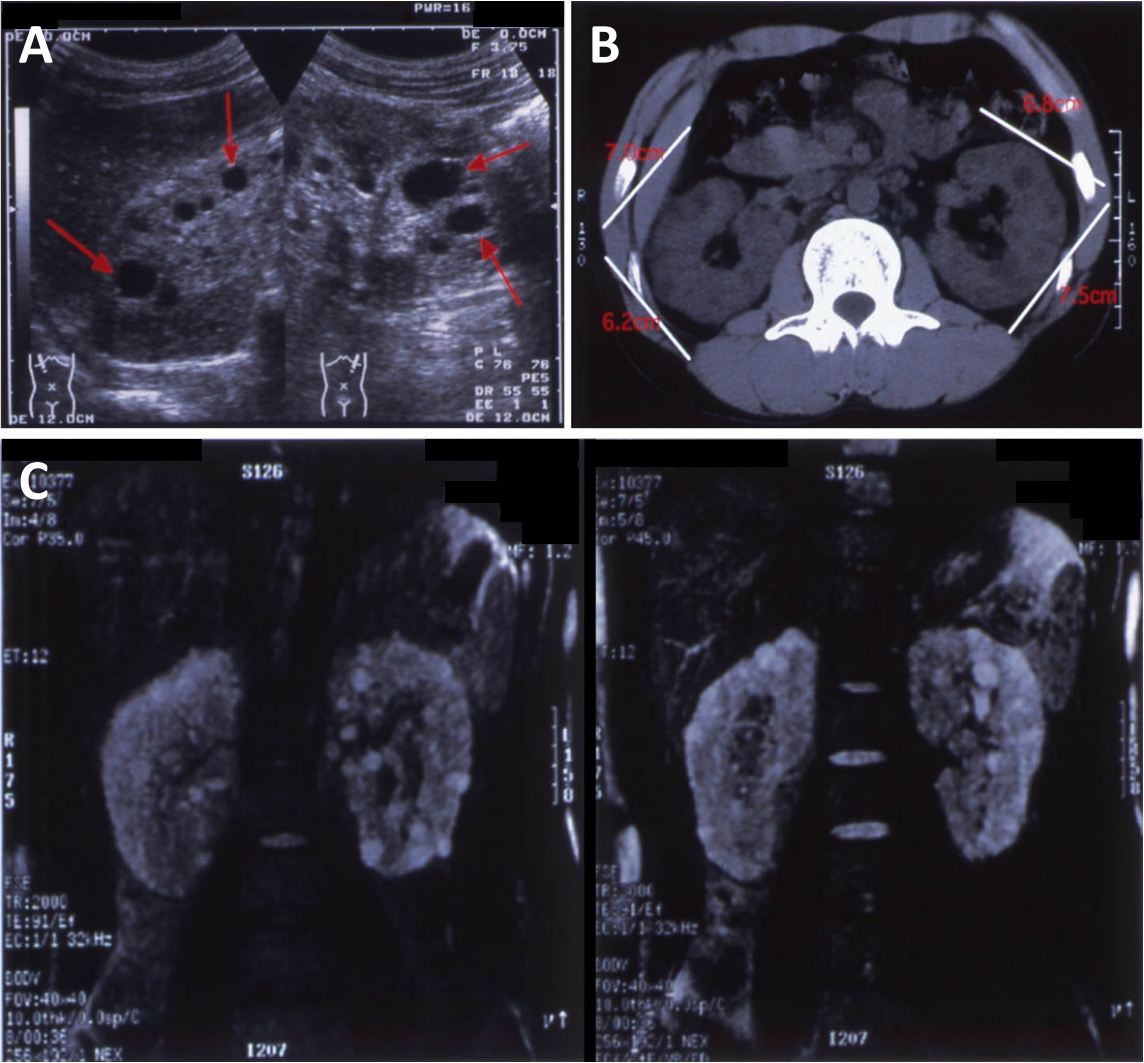
Imaging studies of the kidneys. Both kidneys contain multiple oval lesions, which are up to 20 mm in diameter, are hypoechoic, show low density on computed tomography (CT), and show high signal intensity in T2-weighted magnetic resonance imaging (MRI) (panel **a**: ultrasound image; panel **b**: CT image; panel **c**: T2-weighted MRI)

On admission, the patient was 185 cm tall and weighed 72.4 kg. Blood pressure was 190/100 mmHg. Edema was present in palpebra and in the lower extremities. Laboratory findings were as follows: the erythrocyte count was 3.44 × 10^6^/μL; hemoglobin, 10.9 g/dL; hematocrit, 33.1%; leukocyte count, 7200/μL; platelet count, 268 × 10^3^/μL; total protein concentration, 5.1 g/dL; albumin, 2.2 g/dL; urea nitrogen, 71.0 mg/dL; creatinine, 4.8 mg/dL; and C-reactive protein, 0.1 mg/dL. Urine protein excretion was 17.9 g/day, and urinary sediment contained 11–30 erythrocytes per high-power field. Creatinine clearance was 16 mL/min. Ultrasonography showed multiple cysts up to 2 cm in diameter in both kidneys (Fig. 2a). According to computed tomography (CT) images the right kidney measured 13 × 7 × 6 cm and the left kidney 13 × 7 × 8 cm (Fig. 2b). Bilateral renal cysts showed high signal intensity on T2-weighted magnetic resonance imaging (MRI) (Fig. 2c).

### Second renal biopsy

**Fig. 3:**
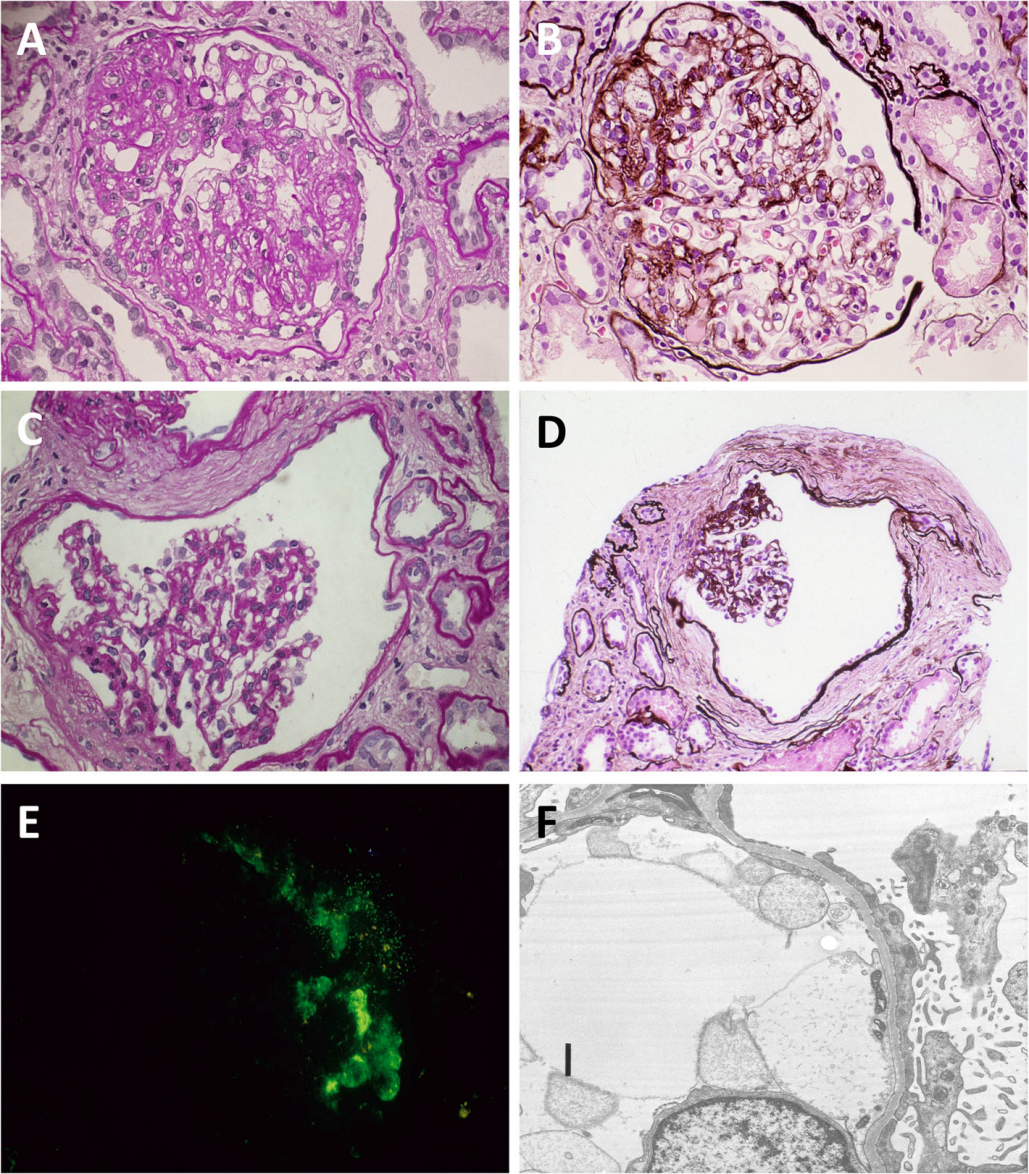
Microscopic findings of the second renal biopsy at the age of 23. Glomerular sclerosis had progressed compared to 4 years ago (panels **a** and **c**: periodic acid-Schiff stain; panels **b** and **d**: periodic acid-methenamine-silver stain). Greater number of segments were adherent to the Bowman’s capsules (panels **a** and **b**), some of which endorsed fibrous crescents (panel **b**). Glomerular collapse and widening of Bowman’s capsules had also progressed and were forming glomerular cysts (panels **c** and **d**). Immunofluorescence microscopy demonstrated IgM deposition in segmental sclerotic lesions of the glomeruli (panel **e**). Electron microscopy revealed diffuse foot process fusion (panel **f**)

A second renal biopsy was performed to reevaluate the pathology behind worsening proteinuria and renal function. Microscopy revealed substantial progression of segmental and global sclerosis of the glomeruli: out of 13 glomeruli in total, four had global sclerosis and seven glomeruli had segmental sclerosis, with a greater number of segments adherent to the Bowman’s capsules compared to the first biopsy (Fig. 3a and b). More glomeruli had collapsed tufts in widened Bowman’s capsules and were forming glomerular cysts (Fig. 3c and d). These glomeruli had fibrous thickening around the Bowman’s capsules. Immunofluorescence microscopy demonstrated deposition of immunoglobulin M in segmental sclerotic lesions (Fig. 3e). Electron microscopy showed diffuse foot process fusion (Fig. 3f). There were no cystic lesions in the tubules or in the interstitium.

### Genetic analysis and diagnosis

The patient’s father and uncle were also found to have PKD. The patient and his father were screened for *PKD1* mutation, which turned out to be positive in both. Thus, he and his father were diagnosed with ADPKD. His father had normal kidney function (creatinine level 0.7 mg/dL), no proteinuria, and small kidney volume regardless of having ADPKD.

The patient’s FSGS was unlikely to be hereditary, because his father did not have proteinuria. The patient had no viral infection, medication use or other factors associated with the development of FSGS. Therefore, his FSGS was likely of primary nature.

### Clinical course

Massive proteinuria was thought to be due to progressing FSGS. Despite 8 months of low-density lipoprotein apheresis in addition to diet therapy, his renal function gradually declined. He started hemodialysis 2 years later, and underwent living donor kidney transplantation subsequently. Proteinuria did not worsen after transplantation and has been consistently below 1 g/day. Twelve years after renal transplantation, his serum creatinine level is stable at around 1.5 mg/dL with oral prednisolone 10 mg, tacrolimus 5 mg, and mycophenolate mofetil 1000 mg per day.

## Discussion and conclusions

This report describes a case of a 23-year-old man with ADPKD with small kidney volume and nephrotic syndrome, which progressed to end-stage renal disease. ADPKD is rarely complicated with nephrotic syndrome. In the Modification of Diet in Renal Disease Study, 200 of the 840 patients enrolled had ADPKD, whose urinary protein excretion was 0.29 ± 0.53 g/day (mean ± standard deviation) in patients with glomerular filtration rate (GFR) of 25–55 mL/min/1.73 m^2^, and 0.46 ± 0.75 g/day in patients with GFR of 13–24 mL/min/1.73 m^2^ [1]. Meanwhile, there have been occasional reports that have documented cases of ADPKD complicated with nephrotic syndrome. Visciano et al. listed 29 reported cases of ADPKD with nephrotic syndrome evaluated by renal histopathological studies and illustrated that FSGS was the most common cause of nephrotic syndrome (6 out of 29 cases) [2]. A literature review by Sumida et al. observed 19 cases of ADPKD with nephrotic syndrome, whose leading cause was FSGS (4 out of 19 cases) manifesting urine protein excretion of 5.8 to 14 g/day [3]. However, due to the rarity of the disease and limited number of reports, histopathology of ADPKD complicated with nephrotic syndrome has not been entirely investigated.

This report illustrates the renal histology of a case of ADPKD accompanied by progressing FSGS, manifesting worsening glomerular segmental sclerosis, glomerular collapse, and glomerular cyst formation. The unique feature of the second biopsy was the progression of glomerular cyst formation. Kriz et al. proposed that glomerular cysts may develop from tubular obstruction due to misdirected filtrate that spreads alongside proximal tubules of nephrons in FSGS patients [4]. According to this theory, the progression of glomerular cysts might have been a feature of worsening FSGS. In addition, glomerular cysts are commonly seen in ADPKD patients [5]. Considering that FSGS is the most common cause of nephrotic syndrome in ADPKD, although it is less common as the cause of nephrotic syndrome in the general population, one hypothesis is that glomerular hyperfiltration due to ADPKD has caused glomerular cyst formation and may be a contributory factor for the development of FSGS in ADPKD patients. While glomerular cysts were prevalent in our case, tubular cysts were not seen in the proximal tubules or in the collecting tubules. Previous research affirms that tubular cysts develop along with progression of PKD, with proximal tubular cysts developing first followed by collecting tubular cysts [6, 7]. This case suggests that glomerular cysts — in contrast to tubular cysts — may be associated with ADPKD with slower volume progression manifesting small kidney volume.

Another notable fact is that the renal function of our patient worsened rapidly and required initiation of renal replacement therapy although the size of the kidney and renal cysts were relatively small for a patient with ADPKD (Fig. 2). The Consortium for Radiologic Imaging Study of PKD (CRISP) investigators illustrated that, the higher the height-adjusted total kidney volume is, the faster the estimated GFR declines [8]. This does not apply to this case, which implies that FSGS was the main cause of the rapid renal impairment in this case. The fact that his father’s renal function was well preserved also supports this idea. This case emphasizes the importance of considering histological evaluation when a patient with ADPKD presents nephrotic-range proteinuria, since it is likely to be caused by other glomerular disease, which may be associated with poor renal prognosis.

In summary, we describe a case of a 23-year-old man with *PKD1*-associated ADPKD with small kidney volume and nephrotic syndrome, which eventually progressed to end-stage renal disease. This is the first report that revealed unique pathological findings of typical focal segmental glomerulosclerosis with remarkable glomerular cyst formation whereas tubular cysts were absent. Glomerular cysts may likely be associated with ADPKD with slower volume progression manifesting small kidney volume, in contrast to tubular cysts which develop along ADPKD progression. Although ADPKD with smaller kidney volume usually shows slower decline in renal function, histological evaluation should be considered when nephrotic-range proteinuria presents in ADPKD in order to look for concomitant glomerular disease that may cause poor renal outcome.

## Data Availability

Further clinical data and images of this case are available from the corresponding authors upon reasonable request.

## Abbreviations

ADPKD: Autosomal dominant polycystic kidney disease
CRISP: Consortium for Radiologic Imaging Study of PKD
CT: Computed tomography
FSGS: Focal segmental glomerulosclerosis
GFR: Glomerular filtration rate
MRI: Magnetic resonance imaging
PKD: Polycystic kidney disease
